# Assessment of the Influence of the Life Cycle of Solar Power Plant Materials and Components on Ecosystem Quality

**DOI:** 10.3390/ma17246028

**Published:** 2024-12-10

**Authors:** Patryk Leda, Grzegorz Szala, Izabela Piasecka

**Affiliations:** 1 Faculty of Mechanical Engineering, Faculty of Mechatronics, Kazimierz Wielki University, Mikołaja Kopernika 1, 85-074 Bydgoszcz, Poland; 2 Faculty of Mechatronics, Kazimierz Wielki University, Mikołaja Kopernika 1, 85-074 Bydgoszcz, Poland; gszala@ukw.edu.pl; 3 Faculty of Mechanical Engineering, Bydgoszcz University of Science and Technology, al. Prof. S. Kaliskiego 7, 85-796 Bydgoszcz, Poland; izabela.piasecka@pbs.edu.pl

**Keywords:** ecosystem, energy, environment, life cycle assessment (LCA), photovoltaic power plant, ReCiPe 2016, renewable energy sources

## Abstract

Currently, silicon is the most often utilized material for photovoltaic cell manufacturing, as it has the potential to convert solar energy directly into electricity. The silicon used in photovoltaic solutions must be highly pure. Large amounts of power, raw materials, and fossil fuels are consumed in the production process. Post-consumer treatment of polymers, materials, and components also requires energy and matter. These processes have a significant influence on the environment. As a result, the primary purpose of this article is to evaluate the influence of a photovoltaic power plant’s material and component life cycle on ecosystem quality. The research focuses on an actual photovoltaic power plant with a capacity of 2 MW located in northern Poland. According to the findings, photovoltaic modules are the part that has the most negative environmental impact, since their manufacturing requires a substantial amount of materials and energy (primarily from conventional sources). Post-consumer management, in the form of recycling after use, would provide major environmental advantages and reduce detrimental environmental consequences throughout the course of the solar power plant’s full life cycle.

## 1. Introduction

### 1.1. Background

The technique of generating electricity using solar power plants is ecologically benign. The process of turning solar energy into electricity does not release dangerous elements into the environment [1,2]. However, it should be noted that the process of manufacturing solar panels is very energy- and material-demanding, resulting in the release of toxic compounds into the atmosphere and ecosystem [1,2,3].

Solar energy is one of the world’s fastest-growing energy technologies, with the average cost of utilizing solar photovoltaics decreasing over time. In recent years, worldwide solar module production volumes have grown at an astonishing yearly rate. At the same time, the average cost of installing solar photovoltaics has steadily fallen since 2010. Global investment in solar photovoltaic energy has expanded dramatically in recent years [4].

Since 2015, global cumulative photovoltaic (PV) capacity has steadily increased. Between 2015 and 2022, the solar market increased by more than 900 gigawatts, moving global markets toward sustainable and distributed energy solutions. In addition, total PV capacity is predicted to surpass 3.5 terawatts by 2027, up more than 2.3 terawatts from 2022 [4].

The enormous growth in community interest in solar systems affects the Earth’s material resources. The growth in manufacturing influences the rise in demand for rare earth raw materials. A lack of sustainable development will have a detrimental impact on ecosystem quality. The depletion of natural resources has recently been a serious concern for countries and organizations such as the United Nations (UN). This is obvious in Chapter 2 of the UN Agenda, which lists the actions that countries must take to protect their natural resources. The depletion of natural resources is regarded as a sustainable development concern. Depletion is a sustainability concern since it degrades present habitats and may have an impact on future generations [5].

The EU’s irresponsible use of natural resources, particularly soil degradation and pollution, contributes significantly to the climate and biodiversity problem. The expenses have already risen to thousands of lives and billions of dollars. Floods, droughts, forest fires, and water shortages are all becoming increasingly common. These events are already having a devastating impact on three-quarters of European countries. Environmental pollution and degradation, droughts, heatwaves, floods, and hitherto identified pests are all contributing to crop and catch reductions. This results not only in losses for farmers and fishermen, but also in higher food costs, which are passed on to consumers [6].

Soil is a living, nonrenewable natural resource that is vital to the environment, economy, and civilization. It is an ecosystem where hundreds of thousands of species interact and collaborate with one another. Soils provide a wide range of ecosystem services that are critical for averting natural catastrophes, addressing climate change, and maintaining food security [1,6]. As a result, materials should be recycled and reused as much as possible during the manufacturing process.

Solar panels can lose up to 20% of their power over time. The highest decline in efficiency is 10% over the first 10 to 12 years and 20% after 25 years. This applies to both the most efficient and the least expensive solar panels on the market. PV panel trash is still classified as normal garbage under regulatory guidelines. The lone exception is at the EU level, where PV panels are classified as electronic trash under the trash Electrical and Electronic Equipment Directive. This regulation, together with other legislative frameworks, governs how PV panel waste is managed. Solar cell producers are obliged by law to follow specific legal criteria and recycling norms to guarantee that solar panels do not harm the environment. This is where solar panel recycling technology began to develop. As a result, if recycling techniques are not developed, 60 million tons of solar panel trash will wind up in landfills by 2050. Because all solar cells include hazardous materials, this would be an extremely unsustainable energy source [1,2,7,8].

As a result, the avoidance of ecological degradation should be prioritized as soon and efficiently as feasible. This might be accomplished by employing the life cycle assessment (LCA) approach, which is a science-based tool for evaluating the numerous environmental burdens, human health consequences, and resource consumption connected with the life of a product, process, or activity. It identifies environmental hot spots and enhances the product system without pushing the load elsewhere. This is accomplished by creating a full inventory of inputs, such as fuels, raw materials, and water, and measuring outputs, such as emissions, products, and byproducts, in relation to their possible environmental consequences across the product’s whole life cycle [3,9,10].

### 1.2. Literature Review

LCA studies are not currently popular. This is a topic that will be widely utilized in solar power plant evaluations in a few or a dozen years. The available research focuses solely on photovoltaic panels. They do not account for all of the components of a solar power plant, including photovoltaic panels, support structures, electrical installations, and inverter stations. The international literature has a few studies that employ the ReCiPe 2016 technology to conduct LCA studies for solar power projects. The studies primarily focus on the influence of solar panel life cycles on global warming potential (GWP), neglecting other negative repercussions for the ecology, the environment around humans and material resources, and land degradation.

Recent research has focused on photovoltaic systems, including greenhouse gas emissions [11], Life Cycle Assessment (LCA) of an integrated PV-ACAES system [12], a comparison of EIA approaches [13], and an LCA of a wind farm power plant [10]. Other research topics include the environmental implications of various energy systems based on LCA [14,15] and the Capacity Optimal Allocation Method [16]. Some LCA studies include analyses of renewable energy sources like wind farms [17], as well as wind turbine components like blades and rotors [18].

Recent studies by other researchers have focused on solar panels rather than full PV power plants, and they have analyzed organic solar cells and perovskite solar cells with transparent graphene electrodes [19], Li et al. re-examined organic and perovskite solar cells [19]. Pisecka et al. [20] and Mao et al. investigated crystalline silicon solar panels [21], Li et al. investigated flexible solar cells [22], Elnozahy et al. investigated the energy efficiency of solar panels [23], and Li et al. analyzed integrated flexible panels. Muteri et al. [24], Ren et al. [25], and Ludin et al. [26] have all provided research on the total impact of solar panels.

There are other studies looking into the overall environmental effect of solar systems [27,28,29,30,31]. Different studies focus on different nations, such as Pakistan [32], New York [33], and Texas [34].

The literature contains research on the recycling of various solar panels [35,36,37]; however, these studies only include the solar panels, not the full photovoltaic power plant.

The themes mentioned in the LCA subject are limited to solar panels. Comprehensive analyses of solar power plants are still missing. However, in a few years, these will be critical studies in the context of sustainable development, since modernization will encompass not only solar panels, but also photovoltaic power plant equipment and electrical systems. As a result, it is required to conduct comprehensive studies of solar power plants, such to those presented in this article.

### 1.3. Research Contribution

Minimizing or eliminating negative consequences is a fundamental problem in sustainable development for renewable energy sources throughout their life cycles. As a result, the primary purpose of the study was to examine the influence of the life cycle of a solar power plant’s materials and components on ecosystem quality.

## 2. Materials and Methods

### 2.1. Object of Analysis

**Figure 1:**
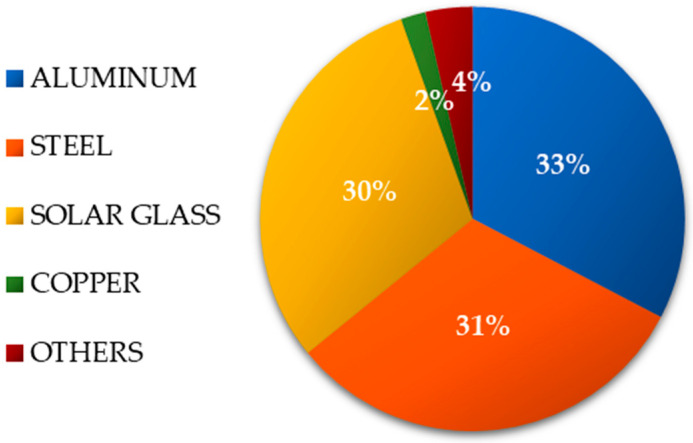
Percentage distribution of component mass in the tested solar energy plant (investor data).

The research object is a solar power plant with a capacity of 2 MW. The tested photovoltaic power plant is located in northern Poland. It generates an average of 1900 to 2200 MWh per year (based on 8 years of operation). The quantity of energy generated from year to year might fluctuate by roughly 10% on average owing to weather unpredictability. For this reason, the reference unit for subsequent studies was assumed to produce 2000 MWh per year. The overall mass of materials, materials, and components in the tested solar power plant is around 300,000 kg (Figure 1). The PV panel support structures included two supports. This was required due to the type of land upon which they were set. The photovoltaic panels face south at a 40-degree slant. The assessed power plant required the installation of 8334 polycrystalline photovoltaic modules with a power of 240 W each. A model with a maximum efficiency of 17.7% was selected.

### 2.2. Methodology of Life Cycle Assessment

The LCA utilized the ReCiPe 2016 model. The ReCiPe approach generates indications for 22 effect groups and three affected locations. Compared to previous models, ReCiPe 2016 offers the most diverse set of impact categories. ReCiPe 2016 is an enhancement of the ReCiPe 2008 model, as well as prior versions like Eco-indicator 99. In contrast to the previous edition, ReCiPe 2016 examines both local and global elements impacting the European region, and as a result, it performs extremely well in the cycle analysis of the presence of renewable energy technology infrastructure [38].

The life cycle assessment technique for products and services is a systematic approach to determining a product’s environmental effect. In this example, a product is defined as a product system that comprises all of the input materials, energy, and transportation required to make the product, as well as its creation and usage until disposal. This approach considers the complete life cycle of a product and assesses all environmental problems connected with it [38].

During the development of the approach, the following structure of the overall LCA study was devised, which consists of four phases:

(a) Goal and scope definition;
(b) Inventory analysis;
(c) Impact assessment;
(d) Interpretation, improvement assessment [38].

The complete approach of LCA analysis is now predominantly incorporated into the following international standards:

(a) EN ISO 14040:2006 Environmental management—life cycle assessment—principles and framework [39];
(b) EN ISO 14044:2006. Environmental management—Life cycle assessment—Requirements and guidelines [40];
(c) ISO/TR 14047:2003. Environmental management—Life cycle impact assessment—Examples of ISO 14,042 implementation [41].

### 2.3. Methodology

The life cycle of a technological renewable energy plant spans from the formulation of the need to be met, through building, manufacture, transportation, sale, and subsequently operation, to the point of post-use management (e.g., recycling or landfill) [2,19].

ReCiPe is one of the Life Cycle Impact Analysis (LCIA) models. It converts emissions and consequences from resource extraction operations into precise values for adopted characteristics. There are two basic methods for determining characteristic factors: at the intermediate point level and at the end point level [42]. The LCA studies were carried out utilizing SimaPro 9.4 software and the ReCiPe 2016 technique. The focus of the analysis was on the characterization process, grouping and weighting using the ReCiPe 2016 approach.

The ReCiPe method’s primary purpose is to reduce a large list of LCI findings to a manageable number of indicator scores. These indicator ratings represent the relative severity of environmental effect categories [43].

ReCiPe 2016 is an upgrade on ReCiPe 2008 and its predecessors, CML 2000 and Eco-indicator 99. The approach is continually revised to reflect new facts and research. The most recent update is being developed by Radboud University [43].

The ReCiPe 2016 project is a joint effort between the Dutch National Institute for Public Health and the Environment (RIVM), Radboud University Nijmegen, the Norwegian University of Science and Technology, and PRé [43].

For our research, we employed the LCA technique based on ISO 14000 [44], ISO 14040 [45], and ISO 14044 [45] standards.

To guarantee the comparability of life cycle assessments, the International Organization for Standardization produced two complimentary standards: ISO 14040 describes the concepts and framework for life cycle assessment, while ISO 14044 specifies the requirements themselves. An LCA in line with the standard normally consists of four phases: identifying the study’s objectives and scope, creating a life cycle inventory (input and output inventory), assessing the effect, and evaluating the results [45].

The ISO 14000 family of standards takes a comprehensive approach to environmental challenges and addresses all topics related to environmental management. They are meant to assist enterprises of all sizes and types in managing the environmental effect of their operations, goods, and services, minimizing negative consequences, and making optimal use of available resources at every level of activity [44].

**Figure 2:**
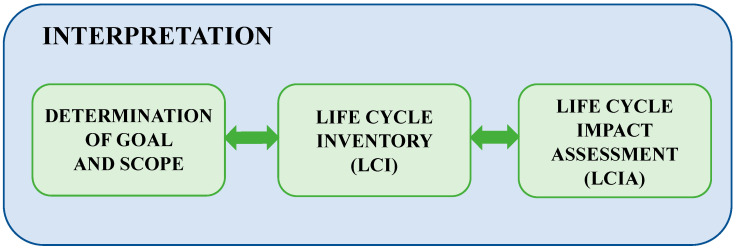
A diagram demonstrating the main processes in LCA analysis [1].

LCA (life cycle assessment) (Figure 2) is the most often used approach for analyzing the environmental life cycle of technical products.

The characterization procedure consists mostly of determining the category index value for LCI findings using characterization parameters. It enables the determination of the proportion of the quantity relevant to the chosen impact category. The acquired result is reported as a numerical value for the index [46].

Characterization characteristics are used to translate LCI findings into a generic category indicator unit. Using these, one may compute the final value of the category indicator, which is the overall influence imposed inside a particular impact category by all LCI emission results categorized in it, or show LCI results in an impact category as a relative value of individual results. Each LCI result is assigned a value for a characterization parameter derived from a specific characterization model. Using this option, all LCI findings for a specific impact category may be transformed to a single unit and totaled [46].

The grouping step gives effect categories to one or more sets in accordance with the study’s goal and scope. Sorting or ranking items based on a certain classification is one example of grouping. This indicates that the grouping method organizes and ranks the effect categories. Impact categories can be aggregated or gathered into an appropriate ranking for simpler understanding; for example, indicators of impact categories with similar qualities can be presented in suitable groups (people health, environmental quality, raw material resources, etc.) [46].

During the LCIA impact assessment, category indicators can be weighted and totaled to determine the weight of the ecological effect. This method is known as weighing (valuation). Weighting is the process of assigning a weight to each effect category so that they may be compared to one another. The most severe consequences are examined first and given the most weight. When constructing a categorization of effects, both regional conditions and the reality of a specific set of individuals for whom the impact is costly are considered (for example, one European’s environmental impact in one year) [46].

## 3. Results

### 3.1. Characterization

Table 1 presents the characterization of the environmental impacts of the solar power plant’s individual unit life cycles. The ReCiPe 2016 model’s impact was evaluated in key categories. Two possibilities for post-consumer management of plastics, materials, and components were considered: landfill and recycling. In terms of the impact of the technical object under study on environmental quality, the life cycle of photovoltaic panels had the highest level of destructive impact, as recorded for the category covering processes causing depletion of water resources and affecting terrestrial ecosystems (2.20 × 10^−1^ species/year). Recycling as a kind of post-consumer management would allow for a reduction in these expenditures during the full life cycle of the technological products under consideration because the recovered polymers, materials, and elements would be reused.

#### 3.1.1. Processes That Cause Depletion of Water Resources and Affect Terrestrial Ecosystems

**Figure 3:**
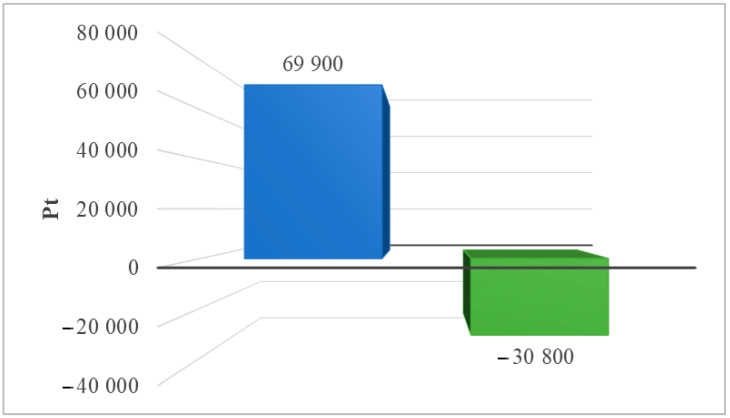
Grouping and weighing the total consequences for the environment of the life cycle of the analyzed photovoltaic power plant in the area of processes causing depletion of water resources, affecting land ecosystems (ReCiPe 2016 model). The method of post-consumer management of materials, materials, and elements is taken into account (unit: Pt) (own research) (blue = landfill; green = recycling).

Table 2 summarizes the environmental impact of the solar power plant’s life cycle, including water resource depletion and impacts on terrestrial ecosystems (ReCiPe 2016 model). Two post-use management scenarios were also taken into consideration. Among the detected compounds influencing terrestrial ecosystems, the use of water in turbines had the greatest detrimental impact (8.94 × 10^5^ Pt during the life cycle, landfill). The adoption of recycling technologies would enable, on the one hand, a reduction in water consumption while also reducing the amount of negative environmental consequences in the investigated region (−1.96 × 10^4^ Pt). Figure 3 shows the total impact of the photovoltaic power plant’s life cycle, including post-use management (landfill, recycling) and the impact of water depletion on terrestrial ecosystems.

**Figure 4:**
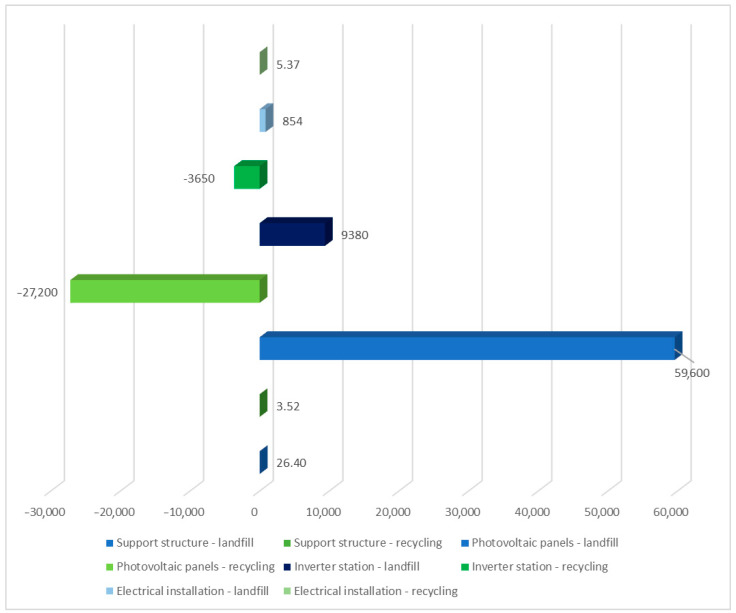
Grouping and weighing the total consequences for the environment of the life cycle of the analyzed photovoltaic power plant in the area of processes causing depletion of water resources, affecting terrestrial ecosystems. The method of post-consumer management of materials, materials, and elements is taken into account (unit: Pt) (own research).

Table 3 summarizes the environmental impact of each solar power plant unit, including water resource depletion and impact on terrestrial ecosystems (ReCiPe 2016 model). Two post-use management situations were taken into consideration. Among the compounds identified as impacting terrestrial ecosystems, the use of water in turbines had the greatest detrimental impact (6.01 × 10^4^ Pt over the life cycle of solar panels, landfill). Recycling techniques can reduce water use and other negative environmental consequences (−2.65 × 10^4^ Pt). Figure 4 summarizes the impact of the solar power plant’s life cycle, including post-use management (landfill, recycling) and the depletion of water resources, which influence terrestrial ecosystems. The largest degree of negative consequences in the studied region is shown in the solar panel life cycle, with post-consumer management in the form of landfill storage.

#### 3.1.2. Emission of Substances Causing Global Warming, Affecting Terrestrial Ecosystems

**Figure 5:**
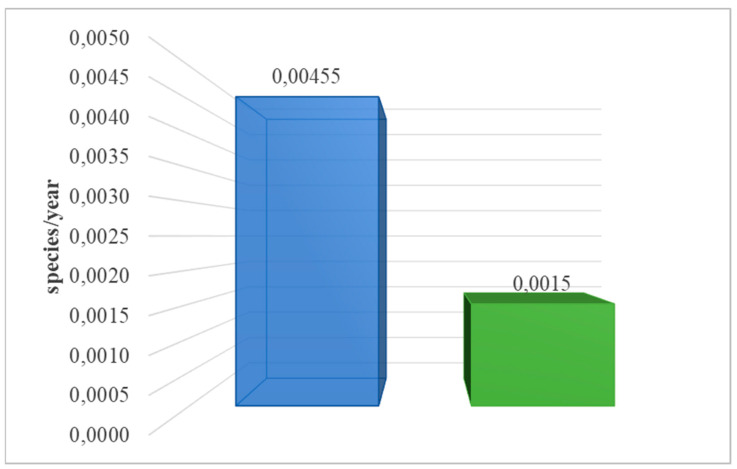
Characterization of the overall consequences of the life cycle of the analyzed photovoltaic power plant for the environment in the area of emissions of substances causing global warming, affecting terrestrial ecosystems (ReCiPe 2016 model). The method of post-consumer management of materials, materials, and elements is taken into account (own research) (blue = landfill; green = recycling).

Table 4 shows the findings gained from analyzing the environmental impacts of the solar power plant’s life cycle. The ReCiPe 2016 model included emissions of chemicals that cause global warming and have an impact on terrestrial ecosystems. Two post-use management scenarios were also considered: landfilling and recycling. Carbon dioxide emissions from fossil sources have the most harmful influence on terrestrial ecosystems (3.45 × 10^−3^ species/year for the life cycle with landfill management). The highest positive effect level was found for the life cycle with recycling management. The recycling process, for example, reduces methane emissions into the environment (−1.41 × 10^−4^ species/year). Methane, which is likewise regarded as a greenhouse gas, is cited far less than CO_2_. However, it is a harmful chemical compound for the environment. During the first 20 years in the atmosphere, one ton of methane has a climatic influence that is 85 times greater than one ton of CO_2_. The energy industry and agriculture are the two main emitters of methane. The energy industry’s primary source of methane emissions is the extraction, transportation, and storage of fossil fuels such as coal, oil, and natural gas. Methane is a major contaminant in the air that we breathe. This can result in illnesses like asthma or emphysema. According to current IPCC reports, worldwide methane emissions should be decreased by around 50% over the next 20 years. No particular reduction measures have been proposed, although the energy sector’s crucial involvement in this respect is highlighted. The essential answer appears to be to transition away from fossil fuels as soon as is feasible and to prevent methane leaks from closed mines and abandoned gas or oil wells. The findings underscore the considerable influence of human activities on air quality and global warming. However, using recycling as a type of post-consumer management would lower the volume of these emissions during the full life cycle of the technological object under consideration because the recovered materials, materials, and elements would not be irretrievably lost (storage), but rather reused. Figure 5 shows the complete effect of a solar power plant’s life cycle, including post-consumer management (landfill, recycling), in terms of emissions that contribute to global warming and harm terrestrial ecosystems.

**Figure 6:**
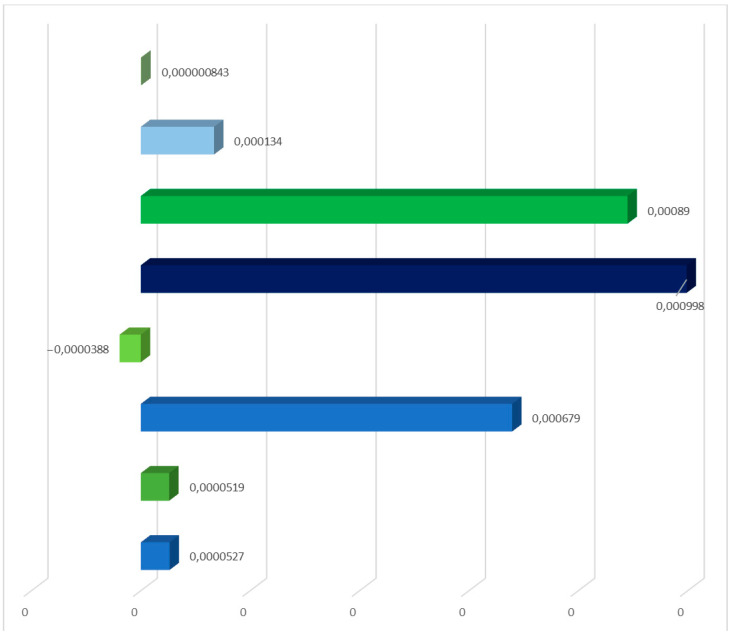
Characterization of the overall consequences for the environment of individual units of the analyzed photovoltaic power plant in the area of emissions of substances causing global warming, affecting terrestrial ecosystems (ReCiPe 2016 model). The method of post-consumer management of materials, materials, and elements is taken into account (unit: species/year) (own research).

Table 5 presents the findings gained from characterizing the environmental impacts of the solar power plant’s individual unit life cycles. The ReCiPe 2016 model included emissions of chemicals that cause global warming and have an impact on terrestrial ecosystems. Two post-use management scenarios were also taken into consideration. Among the identified substances affecting terrestrial ecosystems, the highest level of negative impact is characterized by carbon dioxide emission (from fossil sources) (1.62 × 10^−3^ species/year for the life cycle of photovoltaic panels with management in the form of storage). CO_2_ occurs in and is created by the human body; it is crucial for maintaining the body’s acid–base balance and carrying oxygen, among other things. It is a component of the carbon cycle in nature and a byproduct of combustion and respiration. Excess carbon dioxide in the atmosphere causes, among other things, acidification of the water that absorbs it, which is critical to many marine ecosystems. Above all, as the concentration of this chemical component increases, the greenhouse effect becomes more intense. This not only causes a rise in the Earth’s surface temperature, but also has a number of additional repercussions. Increased carbon dioxide concentration in the breathed air is one of the major causes that might induce a rise in CO_2_ concentration in the blood. It impacts the human body on a daily basis, and most individuals have experienced the detrimental consequences of excessive concentrations of this gas in the air. Increased CO_2_ concentration disrupts human cognitive processes (ranging from basic decisions to complex strategic thinking), and the concentration achieved after several hours in a confined room has a detrimental impact on the efficacy of learning, memory, and attention. Carbon dioxide is a chemical that life on Earth and the functioning of organisms would be impossible without; nevertheless, the issue is not its presence, but rather its rising concentration, which is happening at an alarming rate. The life cycle with recycling management had the most beneficial impact in the assessed area. The recycling procedure allows for a reduction in the emission of, among others, tetrafluoromethane, CFC-14 (−1.23 × 10^−4^ species/year). The findings reveal that human activity has a major influence on decreases in air quality and global warming. Figure 6 shows the complete impact of a solar power plant’s life cycle, including post-consumer management, on global warming emissions and terrestrial ecosystems.

#### 3.1.3. Emission of Substances Causing Soil Acidification

**Figure 7:**
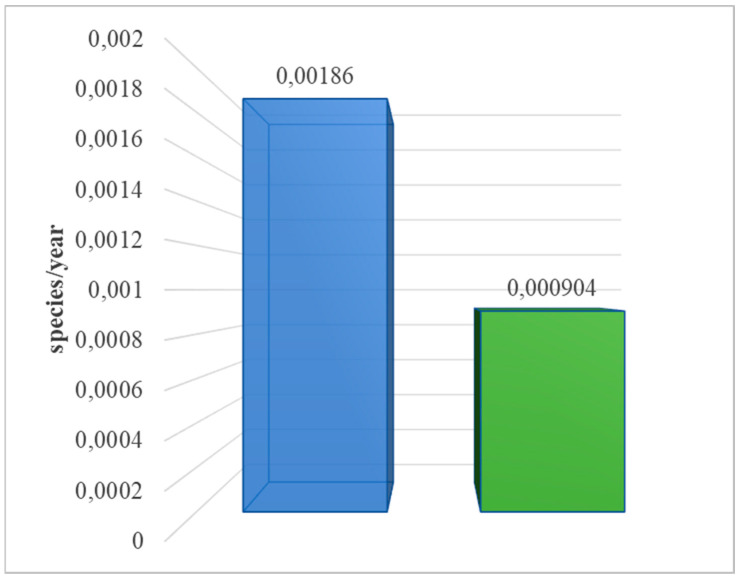
Characterization of the overall consequences of the life cycle of the analyzed photovoltaic power plant for the environment in the area of emissions of substances causing soil acidification (ReCiPe 2016 model), taking into account the method of post-consumer management of materials, materials, and elements (own research) (blue = landfill; green = recycling).

Table 6 summarizes the findings gained from characterizing the environmental impacts of the solar power plant’s life cycle. The ReCiPe 2016 model included emissions of chemicals that cause soil acidification and have an impact on terrestrial ecosystems. Two post-consumer management scenarios for plastics, materials, and components were considered: landfill and recycling. Among the identified compounds influencing terrestrial ecosystems, sulfur dioxide had the most detrimental influence, creating a slew of environmental problems (1.51 × 10^−3^ species/year, landfill). The most favorable impact was noticed over the life cycle of a solar power plant with recycling management. The recycling technique reduced the amount of sulfur dioxide released into the atmosphere, which had a negative, acidifying effect on the ecosystem (7.07 × 10^−4^ species/year). Recycling as a kind of post-consumer management would lower the volume of the aforementioned emissions across the whole life cycle of the technological product under investigation by reusing recovered materials, materials, and elements. Figure 7 shows the complete impact of a solar power plant’s life cycle, including post-consumer management (landfill and recycling) and emissions of compounds that cause soil acidification.

**Figure 8:**
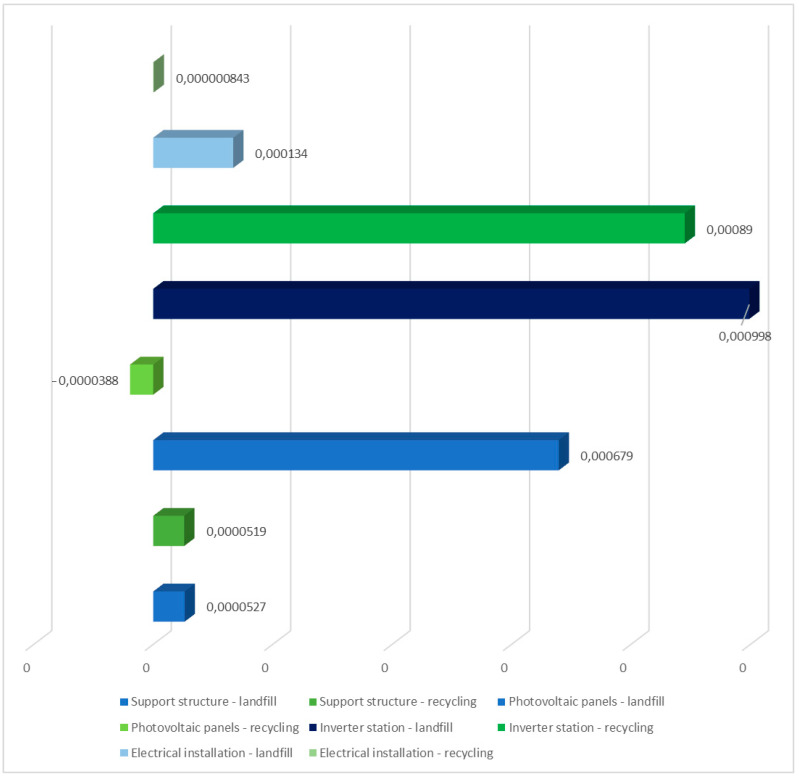
Characterization of the overall consequences for the environment of individual units of the analyzed photovoltaic power plant in the area of emission of substances causing soil acidification (ReCiPe 2016 model), taking into account the method of post-consumer management of materials, materials, and elements (unit: species/year) (own research).

Table 7 shows the findings gained from characterizing the environmental impacts of the solar power plant’s individual unit life cycles. The ReCiPe 2016 model included emissions of chemicals that cause soil acidification and have an impact on terrestrial ecosystems. Two post-use development possibilities were also taken into consideration. Among the detected compounds influencing terrestrial ecosystems, sulfur dioxide had the biggest detrimental impact (5.43 × 10^−4^ species/year during the life cycle of solar panels with landfill). The life cycle with recycling management provided the most positive benefit. Recycling, for example, may significantly reduce sulfur dioxide emissions (−5.56 × 10^−5^ species/year over the PV panel life cycle). The results reveal that human activity has a considerable influence on the decline in air quality or acidification. However, using recycling as a type of post-use management would allow for a reduction in the volume of these emissions during the full life cycle of the tested technological object, because the recovered materials, materials, and elements would not be irretrievably lost (landfill), but rather reused. Figure 8 shows the complete impact of a solar power plant’s life cycle, including post-consumer management (landfill and recycling) and emissions of compounds that cause soil acidification. The life cycles of an inverter station and solar panels with post-consumer management in the form of landfill storage have the most negative environmental impact in the assessed area.

### 3.2. Grouping and Weighting

**Figure 9:**
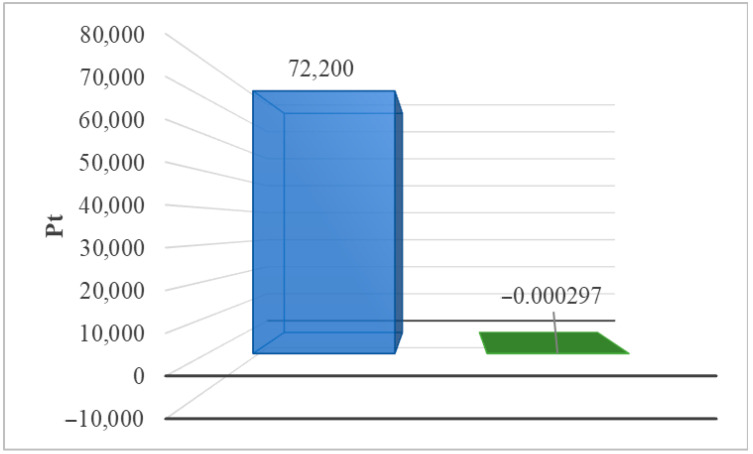
Grouping and weighing the total consequences for the ecosystem of the life cycle of the analyzed photovoltaic power plant (ReCiPe 2016 model), taking into account the method of post-consumer management of materials, materials, and elements (own research) (blue = landfill; green = recycling).

The results of grouping and weighting the consequences for the ecosystem caused by the life cycle of the analyzed photovoltaic power plant are described here. All areas of impact of the ReCiPe 2016 model were taken into account. Two scenarios of post-consumer management of plastics, materials, and elements were also taken into account—landfill or recycling. Recycling, as a form of post-consumer management, would reduce the negative environmental consequences of the technical object under study over its entire life cycle, because the recovered materials, materials, and elements would not be irretrievably lost (landfill), but rather reused. Figure 9 shows the environmental effect of a solar power plant’s life cycle, including post-consumer management options like landfill and recycling.

**Figure 10:**
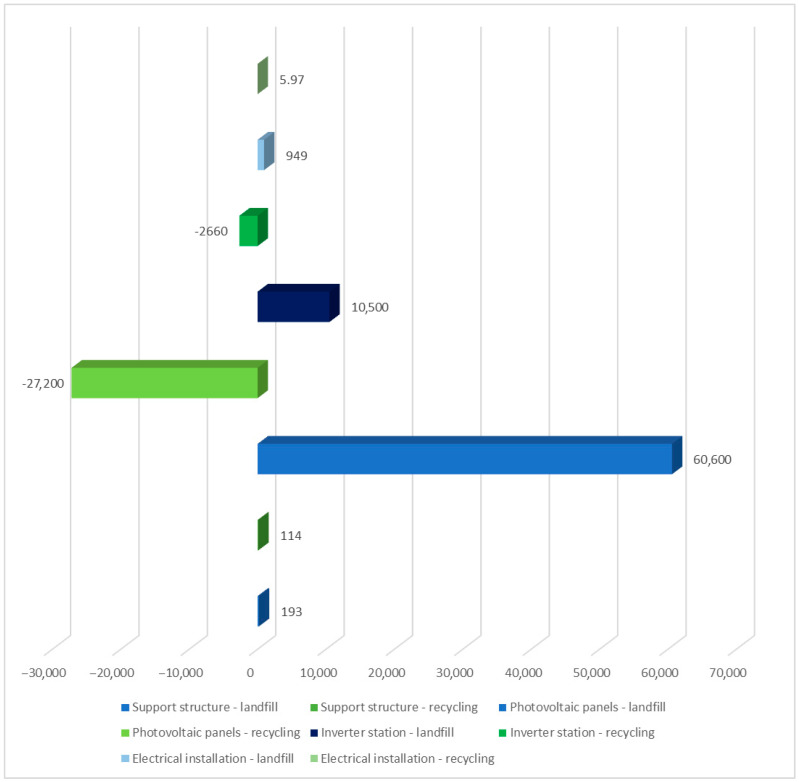
Grouping and weighing the consequences for the ecosystem of the life cycle of individual units of the analyzed photovoltaic power plant in the area of three areas of impact of the ReCiPe 2016 model, taking into account the method of post-consumer management of materials, materials, and elements (unit: Pt) (own research).

The results of grouping and weighing the ecosystem repercussions of the solar power plant’s individual unit life cycle are described here. The ReCiPe 2016 model’s influence was evaluated in all categories. Again, two post-consumer management options were considered: landfill and recycling. The technological object tested had the highest amount of harmful influence on the ecosystem (6.06 × 10^4^ Pt). In this situation, the specified category would also have the maximum degree of positive impact in the assessed respect if recycling were chosen as the mode of post-use management (−2.72 × 10^4^ Pt). The largest degree of negative consequences in the investigated region was shown in the solar panel life cycle, with post-use management in the form of landfills. Figure 10 shows the environmental effect of a solar power plant’s life cycle, including post-consumer management options like landfill and recycling.

**Figure 11:**
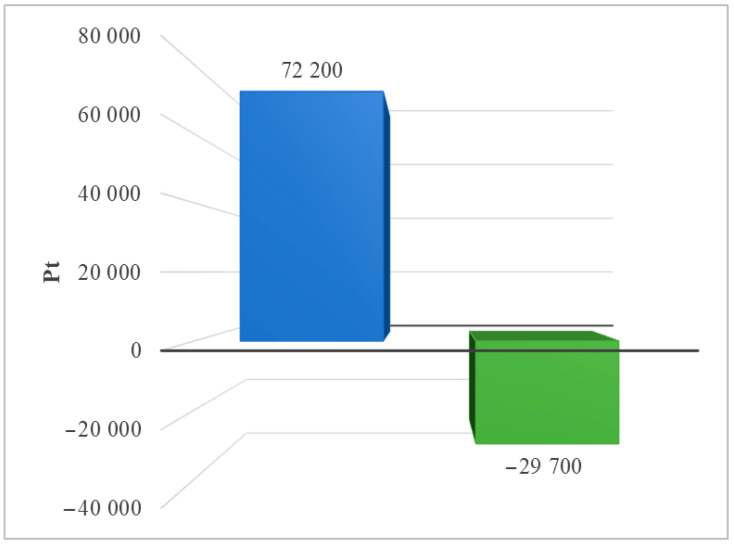
Grouping and weighing the total consequences for the environment of the life cycle of the analyzed photovoltaic power plant in the area of impact on the ecosystem. The ReCiPe 2016 model is used, taking into account the method of post-consumer management of materials, materials, and elements (own research) (blue = landfill; green = recycling).

Table 8 summarizes the findings gained from categorizing and weighing the environmental implications of the solar power plant’s life cycle. The ReCiPe 2016 model took into account negative impacts on ecology. Two post-consumer management scenarios were also taken into consideration. The procedures that used water in turbines had the greatest negative effects (8.9 × 10^4^ Pt during the entire cycle, landfill). These processes had the most beneficial influences on recycling as a type of post-consumer management (−1.96 × 10^4^ Pt). The findings demonstrate the gravity of the issue of depleting drinking water supplies, as well as the major influence of human activities on degrading their quality. Figure 11 shows the complete impact of a solar power plant’s life cycle, including post-consumer management (storage and recycling), on the environment.

**Figure 12:**
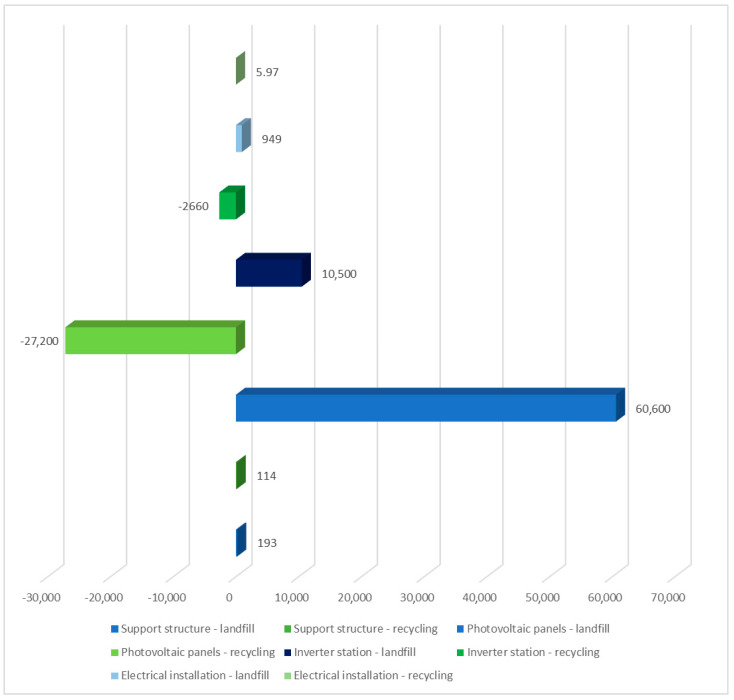
Grouping and weighing the total consequences for the environment of individual units of the analyzed photovoltaic power plant and their impact on the ecosystem. The ReCiPe 2016 model is used, taking into account the method of post-consumer management of materials, materials, and elements (unit: Pt) (own research).

Table 9 shows the results gained from combining and weighing the environmental impacts of the solar power plant’s separate unit life cycles. The ReCiPe 2016 model took into account negative impacts on ecology. Again, two post-consumer management scenarios for plastics, materials, and elements were considered. In this scenario, the largest negative effect was associated with activities that used water in turbines (6.01 × 10^4^ Pt for the life cycle of solar panels, landfill). These methods were also characterized by the maximum amount of beneficial impact in the case of post-consumer recycling (−2.65 × 10^4^ Pt). Figure 12 shows the complete impact of a solar power plant’s life cycle, including post-consumer management (storage and recycling), on the environment. The largest degree of negative consequences in the investigated region is shown in the solar panel life cycle, with post-consumer management in the form of landfill.

## 4. Summary

### 4.1. Conclusions

The primary purpose of sustainable development is to address human needs while also considering future generations’ demands. The socioeconomic growth of highly developed nations involves rapid social and economic development while also enhancing the population’s quality of life and the environment.

The analysis of the actual case study revealed that the solar power plant’s life cycle is consistent with the principles of sustainable development. However, it is vital to implement improvement strategies aimed at decreasing the negative and increasing the positive influence on the environment.

The study of all environmental impact categories included in the ReCiPe 2016 model found that the greatest number of possible negative environmental effects throughout the life cycle of the investigated PV power plant occurred for emissions of compounds that have a negative influence on ecosystems.

The life cycles of four sets of photovoltaic power plant components (support structures, solar panels, inverter station, and electrical installation) were examined. The life cycle of solar panels had the most negative environmental effect, with post-consumer management in the form of landfill. At the same time, it was recognized that the employment of recycling methods in this situation would have the most beneficial environmental impact when compared to other sets of power plant components. This is linked, among other things, to the high energy and material consumption of solar cell production processes.

The damage to the ecology was mostly caused by the release of pollutants that harm the ecosystem into the atmosphere. Pollutants are emitted mostly during the manufacturing of polymers, materials, and power plant components, as well as the burning of conventional fuels to provide the electricity required at this stage of production.

Achieving the research goal allows us to consider the application of the adopted methods as correct and assess them as correct. The analysis performed herein allows us to assess the positive and negative impacts of the photovoltaic power plant’s life cycle in the context of the key area of the ecosystem.

### 4.2. Main Recommendations

The LCA life cycle analysis approach can be used for any technological device or item that will be made and later used. The LCA technique enables the prediction of negative environmental and ecological consequences throughout the design phase. Furthermore, post-consumer management with two management procedures (landfill and recycling) reveals which method, such as a solar power plant, may provide better outcomes after usage.

Recycling, as a kind of post-consumer management, serves to lessen the negative influence on the environment and ecosystem by lowering toxic compound emissions and reusing some resources throughout the manufacturing process. The offered thesis is supported by the research findings mentioned in the paper.

### 4.3. Extending the Scope of Research

The study of the provided hydroelectric power plant utilizing the ReCiPe 2016 approach can be expanded to cover other areas of influence. The ReCiPe 2016 approach is quite thorough. Future studies might examine issues such as depletion of water resources, depletion of material resources, depletion of fossil resources, emissions to the human environment, and many more topics covered by the provided research technique.

## Figures and Tables

**Table 1:** Characterization of the consequences of the life cycle of individual units of the analyzed photovoltaic power plant for the ReCiPe 2016 model, taking into account the method of post-consumer management of materials, components, and elements (unit: species/year) (own research).

No	Element of a Technical Object	Support Structure		Photovoltaic Panels		Inverter Station		Electrical Installation	
	Form of Post- Consumer Development	Landfill	Recycling	Landfill	Recycling	Landfill	Recycling	Landfill	Recycling
	Impact Category								
1	Global warming, Terrestrial ecosystems	3.79 × 10^−4^	2.69 × 10^−4^	2.25 × 10^−3^	−2.83 × 10^−4^	1.86 × 10^−3^	1.51 × 10^−3^	6.59 × 10^−5^	4.14 × 10^−7^
2	Global warming, Freshwater ecosystems	1.03 × 10^−8^	7.35 × 10^−9^	6.13 × 10^−8^	−7.73 × 10^−9^	5.07 × 10^−8^	4.13 × 10^−8^	1.80 × 10^−9^	1.13 × 10^−11^
3	Ozone formation, Terrestrial ecosystems	3.44 × 10^−5^	3.39 × 10^−5^	2.09 × 10^−4^	1.24 × 10^−5^	2.58 × 10^−4^	2.28 × 10^−4^	2.16 × 10^−5^	1.36 × 10^−7^
4	Terrestrial acidification	5.27 × 10^−5^	5.19 × 10^−5^	6.79 × 10^−4^	−3.88 × 10^−5^	9.98 × 10^−4^	8.90 × 10^−4^	1.34 × 10^−4^	8.43 × 10^−7^
5	Freshwater eutrophication	1.10 × 10^−4^	3.24 × 10^−5^	2.88 × 10^−4^	2.09 × 10^−4^	3.24 × 10^−4^	3.11 × 10^−4^	2.48 × 10^−5^	1.56 × 10^−7^
6	Marine eutrophication	7.96 × 10^−8^	4.46 × 10^−9^	1.34 × 10^−7^	1.29 × 10^−7^	1.84 × 10^−7^	1.61 × 10^−7^	1.11 × 10^−8^	6.98 × 10^−11^
7	Terrestrial ecotoxicity	1.75 × 10^−6^	1.74 × 10^−6^	3.30 × 10^−5^	1.60 × 10^−5^	3.15 × 10^−4^	3.12 × 10^−4^	6.66 × 10^−5^	4.19 × 10^−7^
8	Freshwater ecotoxicity	1.99 × 10^−5^	4.01 × 10^−6^	9.32 × 10^−5^	8.57 × 10^−5^	1.56 × 10^−4^	1.52 × 10^−4^	8.66 × 10^−6^	5.45 × 10^−8^
9	Marine ecotoxicity	4.03 × 10^−6^	8.58 × 10^−7^	2.12 × 10^−5^	1.95 × 10^−5^	3.25 × 10^−5^	3.17 × 10^−5^	1.94 × 10^−6^	1.22 × 10^−8^
10	Land use	1.49 × 10^−5^	1.40 × 10^−5^	1.22 × 10^−4^	4.54 × 10^−5^	2.20 × 10^−4^	2.09 × 10^−4^	2.85 × 10^−5^	1.79 × 10^−7^
11	Water consumption, Terrestrial ecosystem	9.76 × 10^−5^	1.30 × 10^−5^	2.20 × 10^−1^	−1.01 × 10^−1^	3.47 × 10^−2^	−1.35 × 10^−2^	3.16 × 10^−3^	1.99 × 10^−5^
12	Water consumption, Aquatic ecosystems	4.78 × 10^−9^	9.95 × 10^−10^	9.85 × 10^−6^	−4.50 × 10^−6^	1.56 × 10^−6^	−6.00 × 10^−7^	1.41 × 10^−7^	8.87 × 10^−10^

**Table 2:** Grouping and weighing the consequences for the environment of the life cycle of the analyzed photovoltaic power plant in the area of the impact of processes causing depletion of water resources, affecting terrestrial ecosystems (ReCiPe 2016 model). The method of post-consumer management of materials, materials, and elements is taken into account (unit: Pt) (own research).

No	Element of a Technical Object	Photovoltaic Power Plant		
	Waste Scenario		Landfill	Recycling
	Substance	Emission Area		
1	Water	Water	−1.96 × 10^4^	−1.96 × 10^4^
2	Water, cooling, unspecified natural origin	Raw materials	2.29 × 10^2^	1.63 × 10^2^
3	Water, lake	Raw materials	1.19 × 10^0^	1.74 × 10^0^
4	Water, river	Raw materials	3.25 × 10^1^	2.62 × 10^1^
5	Water, turbine use, unspecified natural origin	Raw materials	8.91 × 10^4^	−1.14 × 10^4^
6	Water, unspecified natural origin	Raw materials	5.80 × 10^1^	5.66 × 10^1^
7	Water, well	Raw materials	6.06 × 10^0^	6.75 × 10^0^
8	Remaining substances	Raw materials	2.32 × 10^1^	1.02 × 10^1^
**TOTAL**			**6.99** × **10^4^**	**−3.08** × **10^4^**

**Table 3:** Grouping and weighing the consequences for the environment of the life cycle of individual units of the analyzed photovoltaic power plant in the area of processes causing depletion of water resources, affecting terrestrial ecosystems (ReCiPe 2016 model). The method of post-consumer management of materials, materials, and elements is taken into account (unit: Pt) (own research).

No	Element of a Technical Object	Support Structure		Photovoltaic Panels		Inverter Station		Electrical Installation	
	Form of Post- Consumer Development	Landfill	Recycling	Landfill	Recycling	Landfill	Recycling	Landfill	Recycling
	Impact Category								
1	Water	−3.06 × 10^2^	−3.05 × 10^2^	−6.12 × 10^2^	−6.80 × 10^2^	−1.86 × 10^4^	−1.86 × 10^4^	−7.76 × 10^1^	−4.88 × 10^−1^
2	Water, cooling, unspecified natural origin	3.84 × 10^0^	3.87 × 10^0^	5.51 × 10^1^	−4.78 × 10^0^	1.69 × 10^2^	1.64 × 10^2^	8.40 × 10^−1^	5.28 × 10^−3^
3	Water, lake	7.81 × 10^−3^	8.57 × 10^−3^	×	×	1.18 × 10^0^	1.73 × 10^0^	×	×
4	Water, river	1.80 × 10^−1^	1.75 × 10^−1^	6.08 × 10^0^	×	2.54 × 10^1^	2.60 × 10^1^	8.47 × 10^−1^	5.33 × 10^−3^
5	Water, turbine use, unspecified natural origin	3.25 × 10^2^	3.03 × 10^2^	6.01 × 10^4^	−2.65 × 10^4^	2.77 × 10^4^	1.48 × 10^4^	9.28 × 10^2^	5.84 × 10^0^
6	Water, unspecified natural origin	7.17 × 10^−1^	7.05 × 10^−1^	×	×	5.59 × 10^1^	5.59 × 10^1^	1.37 × 10^0^	8.62 × 10^3^
7	Water, well	1.08 × 10^−1^	2.20 × 10^−1^	×	×	5.81 × 10^0^	6.53 × 10^0^	1.44 × 10^−1^	9.06 × 10^4^
8	Remaining substances	7.37 × 10^−2^	5.51 × 10^−3^	8.94 × 10^0^	2.77 × 10^0^	1.36 × 10^1^	7.47 × 10^0^	5.38 × 10^−1^	3.38 × 10^3^
	**Total**	**2.64** × **10^1^**	**3.52** × **10^0^**	**5.96** × **10^4^**	**−2.72** × **10^4^**	**9.38** × **10^3^**	**−3.65** × **10^3^**	**8.54** × **10^2^**	**5.37** × **10^0^**

**Table 4:** Characterization of the consequences of the life cycle of the analyzed photovoltaic power plant for the environment in the area of emissions of substances causing global warming, affecting terrestrial ecosystems (ReCiPe 2016 model). The method of post-consumer management of materials, materials, and elements is taken into account (unit: species/year) (own research).

No	Element of a Technical Object	Photovoltaic Power Plant		
	Waste Scenario		Landfill	Recycling
	Substance	Emission Area		
1	Carbon dioxide, fossil	Air	3.45 × 10^−3^	1.18 × 10^−3^
2	Carbon dioxide, land transformation	Air	1.00 × 10^−5^	9.97 × 10^−6^
3	Dinitrogen monoxide	Air	5.35 × 10^−5^	3.31 × 10^−5^
4	Ethane, haxafluoro-, HFC-116	Air	5.62 × 10^−5^	−2.63 × 10^−5^
5	Hydrocarbons, chlorinated	Air	1.20 × 10^−7^	4.16 × 10^−8^
6	Methane, biogenic	Air	3.17 × 10^−4^	2.35 × 10^−4^
7	Methane, chlorodifluoro-, HCFC-22	Air	2.81 × 10^−7^	4.52 × 10^−7^
8	Methane, dichlorodifluoro-, CFC-12	Air	2.37 × 10^−7^	2.37 × 10^−7^
9	Methane, fossil	Air	3.03 × 10^−4^	1.60 × 10^−4^
10	Methane, tetrafluoro-, CFC-14	Air	3.02 × 10^−4^	−1.41 × 10^−4^
11	Sulfur hexafluoride	Air	5.30 × 10^−5^	4.47 × 10^−5^
12	Remaining substances	x	5.75 × 10^−7^	2.00 × 10^−8^
**TOTAL**			**4.55 × 10^−3^**	**1.50 × 10^−3^**

**Table 5:** Characterization of the consequences of the life cycle of individual units of the analyzed photovoltaic power plant for the environment in the area of emissions of substances causing global warming, affecting terrestrial ecosystems (ReCiPe 2016 model). The method of post-consumer management of materials, materials, and elements is taken into account (unit: species/year) (own research).

No	Element of a Technical Object	Support Structure		Photovoltaic Panels		Inverter Station		Electrical Installation	
	Form of Post- Consumer Development	Landfill	Recycling	Landfill	Recycling	Landfill	Recycling	Landfill	Recycling
	Impact Category								
1	Carbon dioxide, fossil	2.38 × 10^−4^	2.36 × 10^−4^	1.62 × 10^−3^	−3.06 × 10^−4^	1.54 × 10^−3^	1.25 × 10^−3^	4.75 × 10^−5^	2.99 × 10^−7^
2	Carbon dioxide, land transformation	1.76 × 10^−7^	1.76 × 10^−7^	4.29 × 10^−7^	4.29 × 10^−7^	9.36 × 10^−6^	9.36 × 10^−6^	3.61 × 10^−8^	2.27 × 10^−10^
3	Dinitrogen monoxide	1.06 × 10^−6^	8.79 × 10^−7^	1.56 × 10^−5^	−8.15 × 10^−7^	3.54 × 10^−5^	3.30 × 10^−5^	1.42 × 10^−6^	8.93 × 10^−9^
4	Ethane, haxafluoro-, HFC-116	x	x	4.88 × 10^−5^	−2.29 × 10^−5^	7.40 × 10^−6^	−3.38 × 10^−6^	1.69 × 10^−8^	1.06 × 10^−10^
5	Hydrocarbons, chlorinated	x	x	x	4.08 × 10^−8^	x	x	1.20 × 10^−7^	7.55 × 10^−10^
6	Methane, biogenic	1.02 × 10^−4^	1.32 × 10^−7^	1.68 × 10^−4^	1.68 × 10^−4^	3.48 × 10^−5^	6.67 × 10^−5^	1.17 × 10^−5^	7.36 × 10^−8^
7	Methane, chlorodifluoro-, HCFC-22	2.81 × 10^−7^	2.81 × 10^−7^	x	x	x	1.71 × 10^−7^	x	x
8	Methane, dichlorodifluoro-, CFC-12	x	x	2.37 × 10^−7^	2.37 × 10^−7^	x	x	x	x
9	Methane, fossil	3.63 × 10^−5^	3.12 × 10^−5^	1.20 × 10^−4^	2.96 × 10^−6^	1.42 × 10^−4^	1.26 × 10^−4^	4.93 × 10^−6^	3.10 × 10^−8^
10	Methane, tetrafluoro-, CFC-14	x	x	2.62 × 10^−4^	−1.23 × 10^−4^	3.97 × 10^−5^	−1.80 × 10^−5^	9.68 × 10^−8^	6.09 × 10^−10^
11	Sulfur hexafluoride	1.79 × 10^−7^	1.77 × 10^−7^	5.73 × 10^−6^	−1.41 × 10^−6^	4.70 × 10^−5^	4.59 × 10^−5^	1.10 × 10^−7^	6.92 × 10^−10^
12	Remaining substances	2.22 × 10^−8^	2.09 × 10^−7^	2.89 × 10^−7^	−4.68 × 10^−8^	2.61 × 10^−7^	4.59 × 10^−8^	2.97 × 10^−9^	1.87 × 10^−11^
	**Total**	**3.79 × 10^−4^**	**2.69 × 10^−4^**	**2.25 × 10^−3^**	**−2.83 × 10^−4^**	**1.86 × 10^−3^**	**1.51 × 10^−3^**	**6.59 × 10^−5^**	**4.14 × 10^−7^**

**Table 6:** Characterization of the consequences of the life cycle of the analyzed photovoltaic power plant for the environment in the area of emissions of substances causing soil acidification (ReCiPe 2016 model). The method of post-consumer management of materials, materials, and elements is taken into account (unit: species/year) (own research).

No	Element of a Technical Object	Photovoltaic Power Plant		
	Waste Scenario		Landfill	Recycling
	Substance	Emission Area		
1	Ammonia	Air	4.47 × 10^−5^	2.69 × 10^−5^
2	Nitrogen oxides	Air	2.92 × 10^−4^	1.51 × 10^−4^
3	Sulfur dioxide	Air	1.51 × 10^−3^	7.07 × 10^−4^
4	Sulfur oxides	Air	6.60 × 10^−7^	6.60 × 10^−7^
5	Sulfur trioxide	Air	1.86 × 10^−5^	1.86 × 10^−5^
6	Sulfuric acid	Air	3.84 × 10^−7^	3.91 × 10^−7^
7	Remaining substances	x	2.38 × 10^−8^	6.28 × 10^−7^
**TOTAL**			**1.86 × 10^−3^**	**9.04 × 10^−4^**

**Table 7:** Characterization of the consequences of the life cycle of individual units of the analyzed photovoltaic power plant for the environment in the area of emission of substances causing soil acidification (ReCiPe 2016 model), taking into account the method of post-consumer management of materials, materials, and elements [unit: species/year] (Emission Area: Air) (own research).

No	Element of a Technical Object	Support Structure		Photovoltaic Panels		Inverter Station		Electrical Installation	
	Form of Post- Consumer Development	Landfill	Recycling	Landfill	Recycling	Landfill	Recycling	Landfill	Recycling
	Impact Category								
1	Ammonia	1.91 × 10^−6^	1.87 × 10^−6^	1.80 × 10^−5^	9.89 × 10^−6^	1.63 × 10^−5^	1.51 × 10^−5^	8.46 × 10^−6^	5.32 × 10^−8^
2	Nitrogen oxides	1.64 × 10^−5^	1.61 × 10^−5^	1.18 × 10^−4^	6.62 × 10^−6^	1.45 × 10^−4^	1.28 × 10^−4^	1.21 × 10^−5^	7.61 × 10^−8^
3	Sulfur dioxide	3.43 × 10^−5^	3.39 × 10^−5^	5.43 × 10^−4^	−5.56 × 10^−5^	8.18 × 10^−4^	7.28 × 10^−4^	1.13 × 10^−4^	7.11 × 10^−7^
4	Sulfur oxides	3.13 × 10^−8^	3.13 × 10^−8^	1.48 × 10^−7^	1.48 × 10^−7^	4.81 × 10^−7^	4.81 × 10^−7^	x	x
5	Sulfur trioxide	x	x	7.22 × 10^−8^	7.22 × 10^−8^	1.85 × 10^−5^	1.85 × 10^−5^	x	x
6	Sulfuric acid	x	x	x	7.49 × 10^−9^	3.84 × 10^−7^	3.84 × 10^−7^	x	x
7	Remaining substances	3.86 × 10^−9^	3.86 × 10^−9^	8.03 × 10^−9^	4.50 × 10^−10^	6.25 × 10^−9^	6.24 × 10^−7^	5.66 × 10^−9^	3.56 × 10^−11^
	**Total**	**5.27 × 10^−5^**	**5.19 × 10^−5^**	**6.79 × 10^−4^**	**−3.88 × 10^−5^**	**9.98 × 10^−4^**	**8.90 × 10^−4^**	**1.34 × 10^−4^**	**8.43 × 10^−7^**

**Table 8:** Grouping and weighing the consequences for the environment of the life cycle of the analyzed photovoltaic power plant in the area of impact on the ecosystem. The ReCiPe 2016 model is used, taking into account the method of post-consumer management of materials, materials, and elements (unit: Pt) (own research).

No	Element of a Technical Object	Photovoltaic Power Plant		
	Waste Scenario		Landfill	Recycling
	Substance	Emission Area		
1	Ammonia	Air	1.06 × 10^2^	9.58 × 10^1^
2	Antimony	Air	1.78 × 10^−1^	6.87 × 10^−2^
3	Antimony	Water	6.91 × 10^−2^	6.40 × 10^−2^
4	Arsenic	Air	2.39 × 10^−1^	3.62 × 10^−3^
5	Arsenic	Water	x	x
6	Barium	Water	x	x
7	Benzene	Air	3.25 × 10^−2^	3.25 × 10^−2^
8	Benzo(a)pyrene	Air	x	x
9	BOD5 (Biological Oxygen Demand)	Water	2.31 × 10^1^	1.25 × 10^1^
10	Cadmium	Air	2.46 × 10^−1^	2.46 × 10^−3^
12	Carbon dioxide, fossil	Air	9.33 × 10^2^	3.19 × 10^2^
13	Carbon dioxide, land transformation	Air	2.58 × 10^0^	2.58 × 10^0^
14	Carbon disulfide	Air	x	x
15	Chromium	Air	1.92 × 10^1^	1.92 × 10^1^
16	Chromium VI	Air	x	x
17	Chromium VI	Water	1.30 × 10^−1^	1.28 × 10^−1^
18	Chromium VI	Soil	x	x
19	COD (Chemical Oxygen Demand)	Water	7.58 × 10^1^	4.38 × 10^1^
20	Copper	Air	7.60 × 10^1^	5.74 × 10^1^
21	Copper	Water	3.86 × 10^1^	3.39 × 10^1^
22	Dinitrogen monoxide	Air	1.03 × 10^1^	9.17 × 10^0^
23	Dioxin, 2,3,7,8 Tetrachlorodibenzo-p-	Air	x	−9.15 × 10^−1^
24	Ethane, hexafluoro-, HFC-116	Air	1.52 × 10^1^	−6.20 × 10^0^
25	Lead	Air	1.94 × 10^0^	1.50 × 10^0^
26	Mercury	Water	x	4.19 × 10^−3^
27	Methane, biogenic	Air	8.58 × 10^1^	6.35 × 10^1^
28	Methane, chlorodifluoro-, HCFC-22	Air	7.59 × 10^−2^	7.59 × 10^−2^
29	Methane, fossil	Air	8.23 × 10^1^	4.25 × 10^1^
30	Methane, tetrafluoro-, CFC-14	Air	8.14 × 10^1^	−3.81 × 10^1^
31	Nickel	Air	5.87 × 10^0^	4.48 × 10^0^
32	Nickel	Water	1.48 × 10^0^	1.40 × 10^0^
33	Nitrogen oxides	Air	2.13 × 10^2^	1.10 × 10^2^
34	NMVOC, non-methane volatile organic compounds	Air	5.05 × 10^0^	4.47 × 10^0^
35	Occupation (total)	Raw materials	6.96 × 10^1^	6.48 × 10^1^
36	Particulates, <2.5 mm	Air	x	x
37	Phosphate	Water	1.03 × 10^2^	9.30 × 10^1^
38	Phosphorus	Water	x	1.15 × 10^−2^
39	Silver	Water	1.06 × 10^0^	1.06 × 10^0^
40	Sulfur dioxide	Air	4.08 × 10^2^	1.91 × 10^2^
41	Sulfur hexafluoride	Air	1.27 × 10^1^	1.24 × 10^1^
	Sulfur oxides	Air	x	x
42	Sulfur trioxide	Air	5.00 × 10^0^	5.00 × 10^0^
43	Thallium	Water	x	x
44	Transformation, from forest (total)	Raw materials	4.41 × 10^1^	3.76 × 10^1^
45	Transformation, from shrub (total)	Raw materials	3.45 × 10^−2^	3.71 × 10^−1^
46	Transformation, to forest (total)	Raw materials	−2.87 × 10^1^	−3.24 × 10^1^
47	Transformation, to shrub (total)	Raw materials	−3.37 × 10^−2^	−3.63 × 10^−1^
48	Vanadium	Air	2.39 × 10^−2^	2.38 × 10^−2^
49	Vanadium	Water	8.11 × 10^−1^	1.59 × 10^0^
50	Water (total)	Water	−1.96 × 10^4^	−1.96 × 10^4^
51	Water, cooling, unspecified natural origin (total)	Raw materials	2.28 × 10^2^	1.64 × 10^2^
52	Water, lake (total)	Raw materials	1.18 × 10^0^	1.73 × 10^0^
53	Water, river (total)	Raw materials	3.15 × 10^1^	2.59 × 10^1^
54	Water, turbine use, unspecified natural origin (total)	Raw materials	8.91 × 10^4^	−1.15 × 10^4^
55	Water, unspecified natural origin (total)	Raw materials	5.80 × 10^1^	5.66 × 10^1^
56	Water, well (total)	Raw materials	6.14 × 10^0^	6.73 × 10^0^
57	Zinc	Air	4.57 × 10^0^	3.69 × 10^0^
58	Zinc	Water	4.42 × 10^1^	3.94 × 10^1^
59	Zinc	Soil	x	x
60	Remaining substances	x	9.58 × 10^1^	6.11 × 10^1^
**TOTAL**			**7.22 × 10^4^**	**−2.97 × 10^4^**

**Table 9:** Grouping and weighing the consequences of the life cycle of individual units of the analyzed photovoltaic power plant for the environment in the area of impact on the ecosystem. The ReCiPe 2016 model is used, taking into account the method of post-consumer management of materials, materials, and elements (unit: Pt) (Emission Area: Air) (own research).

No	Element of a Technical Object	Emission Area	Support Structure		Photovoltaic Panels		Inverter Station		Electrical Installation	
	Form of Post- Consumer Development		Landfill	Recycling	Landfill	Recycling	Landfill	Recycling	Landfill	Recycling
	Impact Category									
1	Ammonia	Air	5.17 × 10^−1^	5.07 × 10^−1^	x	x	1.03 × 10^2^	9.53 × 10^1^	2.29 × 10^0^	1.44 × 10^−2^
2	Antimony	Air	6.42 × 10^−2^	6.42 × 10^−2^	x	x	x	3.78 × 10^−3^	1.14 × 10^−1^	7.17 × 10^−4^
3	Antimony	Water	6.91 × 10^−2^	6.40 × 10^−2^	x	x	x	x	x	x
4	Arsenic	Air	x	x	x	x	x	2.12 × 10^−3^	2.39 × 10^−1^	1.50 × 10^−3^
5	Arsenic	Water	x	x	x	x	x	x	x	x
6	Barium	Water	x	x	x	x	x	x	x	x
7	Benzene	Air	3.25 × 10^−2^	3.25 × 10^−2^	x	x	x	x	x	x
8	Benzo(a)pyrene	Air	x	x	x	x	x	x	x	x
9	BOD5 (Biological Oxygen Demand)	Water	4.32 × 10^0^	2.95 × 10^−1^	1.13 × 10^1^	5.77 × 10^0^	6.85 × 10^0^	6.42 × 10^0^	5.83 × 10^−1^	3.67 × 10^−3^
10	Cadmium	Air	x	x	x	x	x	9.17 × 10^−4^	2.46 × 10^−1^	1.55 × 10^−3^
11	Carbon dioxide, fossil	Air	6.44 × 10^1^	6.39 × 10^1^	4.39 × 10^2^	−8.29 × 10^1^	4.17 × 10^2^	3.38 × 10^2^	1.29 × 10^1^	8.11 × 10^−2^
12	Carbon dioxide, land transformation	Air	4.77 × 10^−2^	4.77 × 10^−2^	x	x	2.53 × 10^0^	2.53 × 10^0^	x	x
13	Carbon disulfide	Air	x	x	x	x	x	x	x	x
14	Chromium	Air	x	x	x	x	1.92 × 10^1^	1.92 × 10^1^	x	x
15	Chromium VI	Air	x	x	x	x	x	x	x	x
16	Chromium VI	Water	1.30 × 10^−1^	1.28 × 10^−1^	x	x	x	x	x	x
17	Chromium VI	Soil	x	x	x	x	x	x	x	x
18	COD (Chemical Oxygen Demand)	Water	1.75 × 10^1^	6.24 × 10^−1^	3.56 × 10^1^	2.66 × 10^1^	2.05 × 10^1^	1.66 × 10^1^	2.18 × 10^0^	1.37 × 10^−2^
10	Copper	Air	3.03 × 10^−1^	3.02 × 10^−1^	6.34 × 10^0^	2.90 × 10^0^	5.46 × 10^1^	5.41 × 10^1^	1.47 × 10^1^	9.25 × 10^−2^
20	Copper	Water	2.39 × 10^0^	8.55 × 10^−2^	1.58 × 10^1^	1.57 × 10^1^	1.88 × 10^1^	1.8 × 10^1^	1.64 × 10^0^	1.03 × 10^−2^
21	Dinitrogen monoxide	Air	2.87 × 10^−1^	2.38 × 10^−1^	x	x	9.58 × 10^0^	8.93 × 10^0^	3.85 × 10^−1^	2.42 × 10^−3^
22	Dioxin, 2,3,7,8 Tetrachlorodibenzo-p-	Air	x	x	x	x	x	−9.15 × 10^−1^	x	x
23	Ethane, hexafluoro-, HFC-116	Air	x	x	1.32 × 10^1^	−6.20 × 10^0^	2.00 × 10^0^	x	x	x
24	Lead	Air	x	1.15 × 10^−2^	x	x	1.48 × 10^0^	1.48 × 10^0^	4.57 × 10^−1^	2.87 × 10^−3^
25	Mercury	Water	x	x	x	x	x	4.19 × 10^−3^	x	x
26	Methane, biogenic	Air	2.77 × 10^1^	3.58 × 10^−2^	4.55 × 10^1^	4.54 × 10^1^	9.40 × 10^0^	1.80 × 10^1^	3.16 × 10^0^	1.99 × 10^−2^
27	Methane, chlorodifluoro-, HCFC-22	Air	7.59 × 10^−2^	7.59 × 10^−2^	x	x	x	x	x	x
28	Methane, fossil	Air	9.83 × 10^0^	8.43 × 10^0^	3.26 × 10^1^	x	3.85 × 10^1^	3.41 × 10^1^	1.33 × 10^0^	8.36 × 10^−3^
29	Methane, tetrafluoro-, CFC-14	Air	x	x	7.07 × 10^1^	−3.32 × 10^1^	1.07 × 10^1^	−4.88 × 10^0^	x	x
30	Nickel	Air	3.44 × 10^−2^	3.42 × 10^−2^	x	x	4.46 × 10^0^	4.43 × 10^0^	1.37 × 10^0^	8.62 × 10^−3^
31	Nickel	Water	9.00 × 10^−2^	3.12 × 10^−2^	x	x	1.39 × 10^0^	1.37 × 10^0^	x	x
32	Nitrogen oxides	Air	1.19 × 10^1^	1.17 × 10^1^	8.60 × 10^1^	4.82 × 10^0^	1.06 × 10^2^	9.34 × 10^1^	8.83 × 10^0^	5.55 × 10^−2^
33	NMVOC, non-methane volatile organic compounds	Air	1.75 × 10^0^	1.74 × 10^0^	x	x	3.02 × 10^0^	2.73 × 10^0^	2.80 × 10^−1^	1.76 × 10^−3^
34	Occupation (total)	Raw materials	3.83 × 10^0^	3.71 × 10^0^	8.61 × 10^0^	1.04 × 10^1^	4.99 × 10^1^	5.06 × 10^1^	7.32 × 10^0^	4.60 × 10^−2^
35	Particulates, <2.5 mm	Air	x	x	x	x	x	x	x	x
36	Phosphate	Water	7.93 × 10^0^	7.82 × 10^0^	3.08 × 10^1^	2.42 × 10^1^	6.00 × 10^1^	6.10 × 10^1^	3.93 × 10^0^	2.47 × 10^−2^
37	Phosphorus	Water	x	1.15 × 10^−2^	x	x	x	x	x	x
38	Silver	Water	x	x	x	x	1.06 × 10^0^	1.06 × 10^0^	x	x
39	Sulfur dioxide	Air	9.28 × 10^0^	9.16 × 10^0^	1.47 × 10^2^	−1.50 × 10^1^	2.21 × 10^2^	1.97 × 10^2^	3.06 × 10^1^	1.92 × 10^−1^
40	Sulfur hexafluoride	Air	4.80 × 10^−2^	4.79 × 10^−2^	x	x	1.27 × 10^1^	1.24 × 10^1^	x	x
41	Sulfur oxides	Air	x	x	x	x	x	x	x	x
42	Sulfur trioxide	Air	x	x	x	x	5.00 × 10^0^	5.00 × 10^0^	x	x
43	Thallium	Water	x	x	x	x	x	x	x	x
44	Transformation, from forest (total)	Raw materials	1.79 × 10^0^	1.78 × 10^0^	8.19 × 10^0^	4.52 × 10^0^	3.28 × 10^1^	3.13 × 10^1^	1.33 × 10^0^	8.35 × 10^−3^
45	Transformation, from shrub (total)	Raw materials	3.45 × 10^−2^	3.17 × 10^−2^	x	x	x	3.39 × 10^−1^	x	x
46	Transformation, to forest (total)	Raw materials	−1.65 × 10^0^	−1.62 × 10^0^	x	−4.92 × 10^0^	−2.59 × 10^1^	−2.59 × 10^1^	−1.20 × 10^0^	−7.55 × 10^−3^
47	Transformation, to shrub (total)	Raw materials	−3.37 × 10^−2^	−3.09 × 10^−2^	x	x	x	−3.32 × 10^−1^	x	x
48	Vanadium	Air	2.39 × 10^−2^	2.38 × 10^−2^	x	x	x	x	x	x
49	Vanadium	Water	8.11 × 10^−1^	8.05 × 10^−1^	x	x	x	7.86 × 10^−1^	x	x
50	Water (total)	Water	−3.05 × 10^2^	−3.06 × 10^2^	−6.12 × 10^2^	−6.81 × 10^2^	−1.86 × 10^4^	−1.86 × 10^4^	−7.74 × 10^1^	−4.87 × 10^−1^
51	Water, cooling, unspecified natural origin (total)	Raw materials	3.35 × 10^0^	3.59 × 10^0^	5.51 × 10^1^	−4.78 × 10^0^	1.69 × 10^2^	1.65 × 10^2^	8.40 × 10^−1^	5.28 × 10^−3^
52	Water, lake (total)	Raw materials	x	x	x	x	1.18 × 10^0^	1.73 × 10^0^	x	x
53	Water, river (total)	Raw materials	1.19 × 10^−1^	1.50 × 10^−1^	6.08 × 10^0^	x	2.45 × 10^1^	2.57 × 10^1^	8.47 × 10^−1^	5.33 × 10^−3^
54	Water, turbine use, unspecified natural origin (total)	Raw materials	3.27 × 10^2^	3.04 × 10^2^	6.01 × 10^4^	−2.65 × 10^4^	2.77 × 10^4^	1.47 × 10^4^	9.28 × 10^2^	5.84 × 10^0^
55	Water, unspecified natural origin (total)	Raw materials	6.89 × 10^−1^	6.85 × 10^−1^	x	x	5.59 × 10^1^	5.59 × 10^1^	1.37 × 10^0^	8.62 × 10^−3^
56	Water, well (total)	Raw materials	1.83 × 10^−1^	1.96 × 10^−1^	x	x	5.81 × 10^0^	6.53 × 10^0^	1.44 × 10^−1^	9.06 × 10^−4^
57	Zinc	Air	2.15 × 10^−2^	2.12 × 10^−2^	x	x	3.74 × 10^0^	3.67 × 10^0^	8.14 × 10^−1^	5.12 × 10^−3^
58	Zinc	Water	2.95 × 10^0^	1.90 × 10^−1^	1.23 × 10^1^	1.21 × 10^1^	2.80 × 10^1^	2.71 × 10^1^	9.80 × 10^−1^	6.16 × 10^−3^
59	Zinc	Soil	x	x	x	x	x	x	x	x
60	Remaining substances		7.66 × 10^−1^	4.21 × 10^−1^	4.42 × 10^1^	1.06 × 10^1^	4.96 × 10^1^	5.00 × 10^1^	1.24 × 10^0^	7.80 × 10^−3^
	**Total**		**1.93 × 10^2^**	**1.14 × 10^2^**	**6.06 × 10^4^**	**−2.72 × 10^4^**	**1.05 × 10^4^**	**−2.66 × 10^3^**	**9.49 × 10^2^**	**5.97 × 10^0^**

## Data Availability

The original contributions presented in the study are included in the article, further inquiries can be directed to the corresponding author.
